# Proteolysis during Tumor Cell Extravasation *In Vitro*: Metalloproteinase Involvement across Tumor Cell Types

**DOI:** 10.1371/journal.pone.0078413

**Published:** 2013-10-23

**Authors:** Evelyn B. Voura, Jane L. English, Hoi-Ying E. Yu, Andrew T. Ho, Patrick Subarsky, Richard P. Hill, Carlo V. Hojilla, Rama Khokha

**Affiliations:** 1 Ontario Cancer Institute, Princess Margaret Hospital, Toronto, Ontario, Canada; 2 Department of Biology, Dominican College, Orangeburg, New York, United States of America; The University of Hong Kong, Hong Kong

## Abstract

To test if proteolysis is involved in tumor cell extravasation, we developed an *in vitro* model where tumor cells cross an endothelial monolayer cultured on a basement membrane. Using this model we classified the ability of the cells to transmigrate through the endothelial cell barrier onto the underlying matrix, and scored this invasion according to the stage of passage through the endothelium. Metalloproteinase inhibitors reduced tumor cell extravasation by at least 35%. Visualization of protease and cell adhesion molecules by confocal microscopy demonstrated the cell surface localization of MMP-2, MMP-9, MT1-MMP, furin, CD44 and α_v_β_3_, during the process of transendothelial migration. By the addition of inhibitors and bio-modulators we assessed the functional requirement of the aforementioned molecules for efficient migration. Proteolytic digestion occurred at the cell-matrix interface and was most evident during the migratory stage. All of the inhibitors and biomodulators affected the transition of the tumor cells into the migratory stage, highlighting the most prevalent use of proteolysis at this particular step of tumor cell extravasation. These data suggest that a proteolytic interface operates at the tumor cell surface within the tumor-endothelial cell microenvironment.

## Introduction

Advances in understanding cancer cell metastasis, particularly the events necessary for directing and enabling metastasizing tumor cell extravasation, have been hindered by the inability to dissect the elements responsible for these processes at the cell-matrix interface of the invading tumor cell. Proteases have long been thought to promote metastasis with supporting evidence being gathered at several indirect levels. However, an understanding of the exact interactions operating during proteolytic processes at the tumor cell surface, as the cell crosses the critical barriers of the endothelium and extracellular matrix (ECM), is still required, particularly in light of the often vital role proteases play in the maintenance of general human homeostasis [1,2].

Secreted matrix metalloproteinases (MMPs), membrane type (MT)-MMPs and serine proteinases are the principal enzymes responsible for ECM degradation [3]. Of particular importance to extravasation are the mechanisms that lead to the generation of the pericellular zone of proteolysis, and the orchestration of molecules that focus it to this location. Activation of plasmin by urokinase plasminogen activator and its receptor, and activation of pro-MMP-2 (gelatinase A) through the assembly of the trimolecular complex (MT1-MMP, MMP-2, and their tissue inhibitor, TIMP-2), are postulated as two key mechanisms for cell surface activation and localization of proteases [4–7]. Processing of MMP-2 depends on prior MT1-MMP activation. It is thought that MT1-MMP is activated by the proprotein convertase furin, although furin-independent activation of MT1-MMP has been reported [8–10]. Golubkov et al. demonstrated the importance of the furin-mediated activation of MT1-MMP for tumorigenicity [11], while others used a small molecule inhibitor of the process to reduce the invasiveness of HT1080 cells [12]. Active furin cycles between the Golgi and the cell surface leading to MT1-MMP activation at both locations [9,13,14]. In addition, the uPA-plasmin system may also contribute to the cell surface activation of pro-MMP-2 [15].

Cell adhesion molecules are also linked to surface proteolysis. The integrin α_v_β_3_ provides an additional means of localizing active MMP-2 to the cell surface [16–18]. Co-localization of α_v_β_3_ and MMP-2 was first observed on angiogenic blood vessels and at the tumor invasive front. This association contributes to the invasion of mesenchymal cells [19]. Leroy-Dudal et al. showed that MMP-2 and α_v_ integrins are important for the invasion endothelial monolayers by ovarian carcinoma cells *in vitro* [20], while Kargozaran et al. suggested that MMP-2 is produced by the endothelium during cancer cell transmigration of an endothelial-basement membrane barrier [21]. Alternatively, it has been reported that MMP-2 activity can guide invasion by cleaving the extracellular matrix, making a route for α_v_β_3_ integrin-mediated cellular motility [22]. α_2_β_1_ is also suggested to be involved in modulating MMP-2 activation at the cell surface via an association of pro-MMP-2 with α_2_β_1_ integrin-bound collagen, to provide an enzyme reserve for subsequent membrane activation [23,24]. 

Additionally, CD44, which is known to promote tumor cell motility and invasion, can anchor active MMP-9 to the cell surface, and has been localized with MMP-9 and MT1-MMP on cellular invadipodia [6,25–33]. It was also shown that a variant of CD44, CD44st, can increase the invasive capacity of the MCF-7 breast cancer cell line, and that this effect involved both MMP-2 and -9 [34]. So, while much evidence suggests that adhesion molecules functionally contribute to the proteolytic interface during metastasis, this association, as well as how it functions in the different stages of the process has yet to be firmly documented and established. 

A crucial step in the metastatic cascade is tumor cell extravasation but, what regulates extravasation and whether proteases are necessary or sufficient for the process remains an open question [35–37]. To begin to address this complex process, we developed a straightforward *in vitro* model to study the transendothelial migration of tumor cells [38–40]. We previously used this system to document the requirement for certain cell adhesion molecules, including α_v_β_3_, for mediating tumor cell interactions with the endothelium during invasion [41,42]. The simplicity of our model proved especially important when attempting to understand the complexity of interactions involving proteolysis during tumor cell transendothelial migration. Since extravasation involves relatively few cells in a process that takes place deep within tissues where many proteinases bathe the extracellular milieu, an *in vitro* system of extravasation offers the potential of asking if proteinases have a role to play during the process in a more controlled background. Using our model we demonstrate that proteolysis is involved in tumor cell extravasation and highlight MT1-MMP and MMP-9 as important enzymes for the migration of different tumor cell types. 

## Materials and Methods

### Cell culture

Commercially available, human microvascular endothelial cells were purchased and maintained in EBM-2 media with 5% FBS and EGM-2 MV SingleQuots supplements and growth factors all from Clonetics/Cambrex Bio Science (Walkersville, Inc.). These were the same cells as used in our previously published work [42]. The commercially available melanoma cell line, WM239, was a gift from Dr. M. Herlyn (The Wistar Institute) and was cultured in RPMI with 10% fetal bovine serum (FBS). These cells were also used in our previous reports [38–42]. MDA-MB231 cells were from ATCC and were cultured in αMEM plus 10% FBS. 

### Modifying reagents

The following were purchased as indicated: aprotinin (Sigma); ilomastat (MPi, AMS Sci.); recombinant tissue inhibitor of metalloproteinase-2 (TIMP-2; PF021-3UG), MT1-MMP Ab (IM39L), and MMP-9 Ab (IM09L) were from Oncogene; Recombinant TIMP-1 was from Dr. A. Docherty (Slough, UK); wild-type and mutant collagenase resistant murine collagen type I from Dr. S. Krane (Charlestown, MA), the furin convertase was from Affinity Bioreagents (RP-062), while the furin inhibiting peptide (N-1505) was from Bachem (Torrance, CA). The hemopexin C domain (HxCD) and collagen-binding domain (CBD) recombinant proteins were prepared as described and were kindly provided by Dr. C.M. Overall (University of British Columbia) [7,23]. The non-blocking mAb PECAM-1.1 against CD31 was from Dr. P. Newman (The Blood Research Institute, Milwaukee). Cyclic RGD and RAD peptides (ICA-4304; PCA-3618-PI; Peptides Int.) were a gift from Dr. C.-H. Siu (University of Toronto). 

### Transendothelial migration assay

Round glass coverslips (12 mm in diameter, 1 mm thick) were coated with 1:8 Matrigel:H_2_O (Beckton Dickinson, Bedford, MA) basement membrane (BM) extracellular matrix (ECM) in a 24-well plate and air-dried overnight. Matrigel was rehydrated in Hanks’ buffered saline solution (HBSS) and the coverslips were transferred to 35-mm dishes. A 200 μL drop containing 1.8 × 10^5^ human microvascular endothelial cells (HMVECs) was plated on the Matrigel-coated coverslips for at least 3 hours so that tight HMVEC monolayers could form. The coverslips were transferred to a 24-well plate and incubated in 350 to 500 μL endothelial growth medium supplemented with 10 ng/mL TNFα (Sigma, St. Louis, MO). These were left overnight at 37°C before use. Transendothelial migration assays were started the following morning after the addition of 3 × 10^4^ fluorescently tagged tumor cells in 25 μL of HMVEC media.

A variety of tags were used to label tumor cells during the assay. This included incubation with 12.5 μM 1,1’-dioctadecyl-3,3’,3’-tetramethylindocarbocyanine perchlorate (DiI; Molecular Probes; 12.5 μM) just prior to addition to endothelial cell monolayers. If orange or blue cell tracker dyes (Molecular Probes) were used to tag the tumor cells, labeling was performed on the evening prior to conducting the assay by incubation in 10 μM of the dye for 1 hour at 37°C. Tagging with Hoechst 33258 (Molecular Probes) was done using 10 μg/mL Hoechst overnight. Modifying reagents were added to the endothelial monolayers 30 minutes prior to the addition of the tumor cells. Monolayer integrity was routinely assessed microscopically before adding the tumor cells.

Epifluorescence microscopy was used to quantify the number of cells at each stage of transmigration. Experiments were done in triplicate and three sets of 15 random fields were scored. 750-1500 cells were scored for each condition. All cell counts were carried out on dual stained (F-actin and orange or blue cell tracker) cancer cells using a 40X objective on a Leitz LABORLUX S fluorescent microscope. Note that Hoechst was typically added to visualize the relative location (focal plane) of both tumor and endothelial cells. F-actin staining served a dual purpose in that it facilitated the assessment of tumor cell migration [38] and the integrity of the endothelial monolayer. Only single or paired cells were scored. Samples were always scored blind by two investigators.

### Immunofluorescence staining

Coverslips were fixed in 3.5% (w/v) paraformaldehyde in phosphate buffered saline (PBS) or ice cold MeOH. Unless indicated, paraformaldehyde fixed cells were permeabilized in a cytoskeleton-stabilizing buffer, pH 6.9, containing, 0.1 M 1,4-piperazinebis (ethanesulfonic) acid, 1 mM EGTA, 0.1 M KOH, 4% (w/v) polyethylene glycol 8000, and 0.1% Triton-X 100. Coverslips were stained in 1:10 dipyrrometheneboron difluoride-conjugated phallacidin (BODIPY-FL phallicidin, Molecular Probes). Strips cut from plastic coverslips (stacked two high) were used as spacers and slides mounted with 72% glycerol in PBS containing 2.5% (w/v) antioxidant 1,4-diazabicycol-[2,2,2]-octane (Sigma). Preparations were sealed with nail enamel. 

Primary antibodies used for immunofluorescence included MT1-MMP Ab (IM57L) from Oncogene. Other antibodies included TIMP-2 pAb, MMP-9 pAb, MMP-2 pAb and clone LM609 mAb against α_v_β_3_ from Chemicon International. The Neomarkers (Freemont, CA) antibody raised against MT1-MMP (1544) was also used. 

For confocal analysis, primary antibodies were diluted 1:100 in PBS. When F-actin was stained, the initial dilution of primary antibodies was supplemented with a 1:10 dilution of either Texas red-conjugated phallicidin (“red actin” stain) or coumarin phalloidin (“blue” actin stain) (Molecular Probes). Endothelial actin was differentiated from tumor cell actin, as tumor cells were always dual stained as described above. Such staining permitted visualization of the entire contour of the tumor cell. The purpose of the actin staining in each experiment was to ensure that the endothelial monolayer was intact. Note, that while each experiment included actin staining, it is not obvious in every image, either because the focal plane was ‘above’ that of the endothelium, or because the signal from the cell tracker dye overwhelmed the weaker actin signal during imaging. Secondary antibodies (Invitrogen/Molecular Probes) were used at 1:200 or 1:300 dilutions. These were either goat-anti-mouse or goat-anti-rabbit conjugated to Alexa 488, 596 or 633. Images were captured using a Zeiss Axiovert 100M or 200 inverted microscope equipped with a 63X oil c-apochromat objective lens or a 63X plan-apochromat objective lens as well as a LSM 510 confocal attachment and/or an LSM 510 META device. 

### Gelatin substrate digestion experiments

For matrix digestion studies, a 1:10 dilution of FITC-conjugated gelatin (fluorogenic) substrate (Molecular Probes) was added to the Matrigel and the transendoethelial migration assay was performed as described above. Two sets of 25 orange tracker-labeled cells in each of seeking, migrating and spreading classes were scored for the extent of substrate fluorescence at the cell contours (- to +++) as described more thoroughly in the Results. A 100X objective with oil immersion was used and images were taken with a Leica WILD modular photomicrographic system 52. All measurements were performed in duplicate by two independent investigators (a total of four experiments). 

For gelatin digestion analyses, 1 mg/mL of FITC-conjugated gelatin (Molecular Probes) was diluted 1:100 in sterile water. A 24-well plate was coated with 100 μL of the labeled gelatin and air-dried overnight. 6 x 10^4^ tumor cells were plated on the gelatin preparation in serum-free media. The cells were left to interact with the gelatin for 3 hours at 37°C. Afterward, the resulting increase in the fluorescence of the fluorogenic substrate due to any cellular digestion of the matrix was measured using a fluorescent microplate reader (TECAN).

To make the 3D optical rotations of the cell surface associated digestion and internalization of the fluorogenic substrate, 1 μm confocal z stack images were acquired using Zeiss LSM 5 software and then Metamorph software (Universal Imaging Corporation) was used to assemble the image stacks into animated movie files.

### Western Blotting

Cell lysates were prepared using an 80% confluent plate of cells grown in 100-mm dishes. The cells were incubated in HMVEC media for 3 hours. The cells were washed 3 times with Hank’s Buffered Saline and then lysed on ice in 1 mL of lysis buffer (10 mM Tris pH 7.5, 5 mM EDTA, 50 mM NaCl, 30 mM Na_4_P_2_O_7_, 1% Triton X-100, 50 mM NaF, 200 μM Na_3_VO_4_, 1 mM phenylmethylsufonylfluroide, 5 μg/mL aprotinin, 1 μg/mL pepstatin A and 2 μg/mL leupeptin). Detergent extracts, normalized to equivalent protein content, were resolved on 8% SDS-PAGE gels under reducing conditions. The proteins were then transferred to Hybond ECL nitrocellulose membranes, which were subsequently blocked with 5% skim milk in Tris buffered saline with 0.1% Tween (TBST) for an hour at room temperature. The blots were then probed using antibodies raised against the β_3_ integrin subunit (181720, Transduction Labs; 1:2500), α-tubulin (CP06, Oncogene; 1:6000) and MT1-MMP (AB815, Chemicon; 1:1000). The dilutions were in TBST with 5% skim milk, and were run overnight at 4°C. The membranes were washed with TBST and then incubated with the appropriate HRP-conjugated secondary antibody for 1 hour at room temperature. The secondary antibody incubation was followed by three washes in TBST. The location of antibody binding was revealed with LumiGLO reagents (New England Biolabs, Mississauga, ON) or Supersignal West Pico chemiluminescent substrate (Pierce).

### Gelatin-based Zymography

Serum-free culture media samples from EC, MDA-MB231 and WM239 cells were collected. To remove any floating cells, supernatants were spun at 2700 rpm for 10 minutes and any resulting pellet was discarded. 5 μL of the resulting cleared media was subjected to SDS-PAGE on 10% polyacrylamide gels containing 0.1% gelatin. After electrophoresis, the gels were maintained in two 30-minute incubations with rinse buffer (2.5% Triton X-100). The gels were then washed briefly with water and then incubated for 24 hours at 37°C in substrate buffer (50mM Tris pH 7.5, 5 mM CaCl_2_). The gels were then stained in Coomassie brilliant blue for 30 minutes at room temperature and destained to reveal any digested bands.

### Statistical analysis

Error bars are standard deviation or standard error of the mean as indicated. Two-tailed Student’s t-tests were performed to assess the significant difference between data sets. Asterisk (*) = p<0.01.

## Results

### Protease inhibitors reduce tumor cell transendothelial migration

Metastatic cancer cells extravasate by passing through the endothelium and degrading the underlying basement membrane and ECM components of the surrounding tissue. This process can be mimicked *in vitro* using a transendothelial migration assay. Previously we used this assay to demonstrate the role of the cell adhesion molecules α_v_β_3_ and L1 during extravasation [42]. Building on these studies, we asked whether MMPs are involved in this process. We examined the extravasation of two well-known metastatic tumor cell lines, MDA-MB231 breast cancer cells and WM239 melanoma cells. 

**Figure 1:**
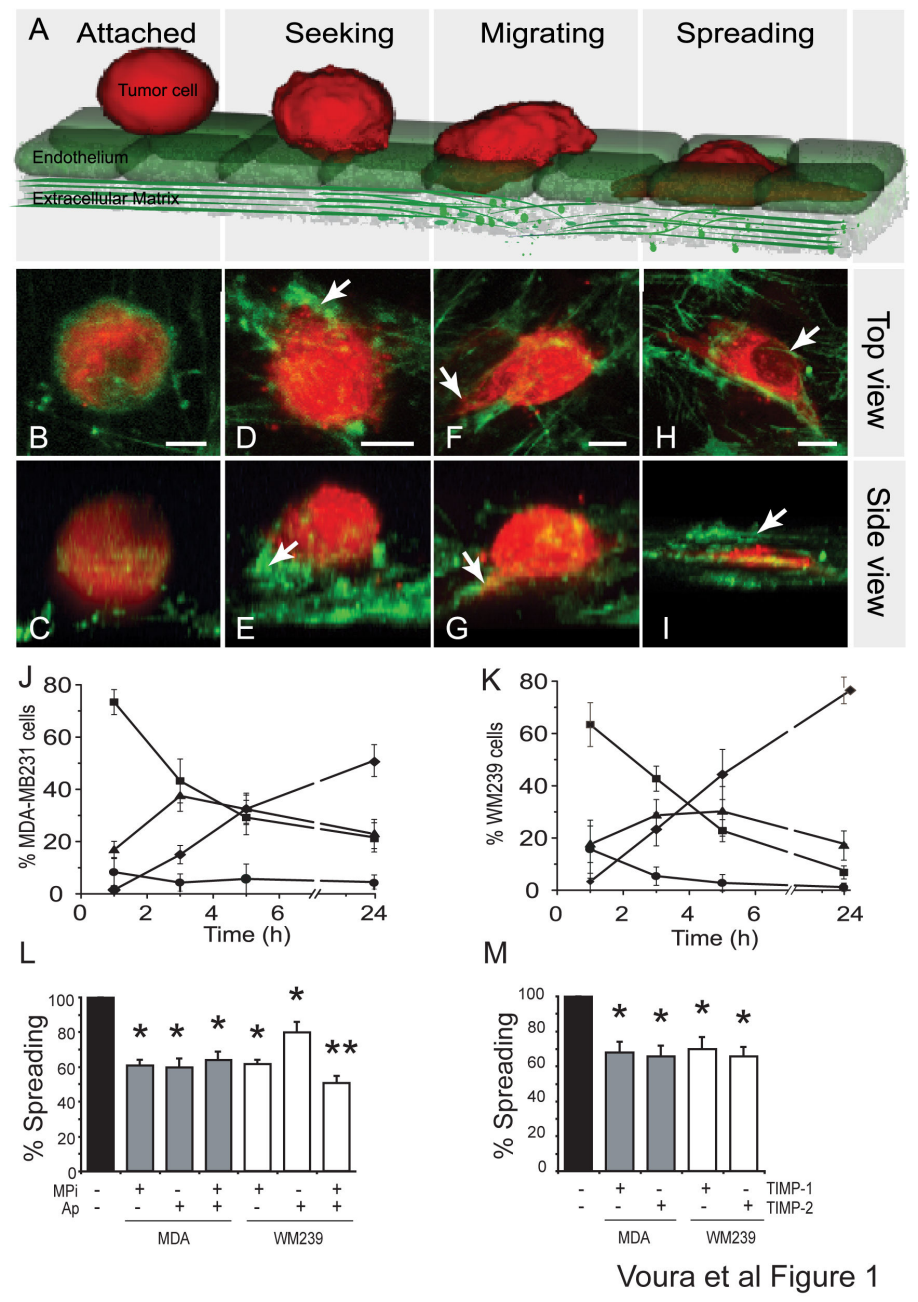
Protease inhibitors reduce tumor cell transendothelial migration. (A) Model of extravasation stages. Three-dimensional confocal rotations or maximum projections of DiI labeled WM238 (red) at stages of extravasation from the top (B, D, F and H) and side (C, E, G, and I). BODIPY FL phallacidin (green) shows actin, and arrows indicate processes. Bars=10 μm. Note that confocal rotations or maximum projections allow for the fluorescence in all the focal planes to be seen in one image. Therefore, the ‘top view’ of the ‘attached’ stage allows for the visualization of actin in both the tumor cells and the endothelial cells in the same image, even if the latter cells are well below the top focal plane of the tumor cell. For reference please also compare with the ‘side view’ panel. Percent attached (●), seeking (■), migrating () and spreading (◆) cells at 1, 3, 5 and 24 hours for MDA-MB231 (J) and WM239 (K). (L) Percent spreading MDA-MB231 (grey bars) and WM239 (white bars) relative to the no inhibitor control (black bar). Proteinase inhibitors MPi (20 μM) and aprotinin (100 U) were used. A * Indicates a significant p-value compared to controls and ** indicates significant p-value relative to both controls and MPi or aprotinin alone. (M) The reduction in spreading cells with recombinant TIMP-1 or TIMP-2 (each at 2 μg/mL).

Based on our previous findings, we divided tumor cell transendothelial migration into four stages (Figure 1A) [38,39]. These steps were first established using confocal microscopic analyses of cellular morphologies and the distribution of actin during the transendothelial migration of fluorescently labeled tumor cells (Figures 1B - 1I; optical rotations/maximum projections showing stacks of all image slices – top view allows for visualization of both tumor and endothelial actin in all focal planes – refer to side view as a comparison). As in previous reports, we translated our confocal observations into morphologies that could be classified by epifluorescence microscopy. ‘Attached’ cells appeared as reflective spheres ‘sitting’ atop the endothelial monolayer (Figures 1B and 1C) ‘Seeking’ cells were also largely spherical, but unlike attached cells, displayed an abundance of ‘bleb-like’ structures on their surface (Figures 1D and 1E). On occasion these blebbing cells extended one or more tiny filopodes into the plane of the endothelium. ‘Migrating’ cells lost their spherical appearance; instead they displayed small circumferentially distributed lamellipodia that formed at, and passed under, the level of the endothelial monolayer giving a ‘fried-egg-like’ appearance. Other migrating cells sent out individual, large lamellipodia that extended beneath the endothelium (arrows; Figures 1F and 1G). ‘Spreading’ cells, on the other hand, were flat, completely extended, and fully or partially covered by the endothelial monolayer as demonstrated by endothelial actin extending over spreading tumor cells (arrow; Figure 1I). Confocal microscopic analysis indicated that endothelial cells were active even at the earliest stages of tumor cell extravasation, changing shape and responding to the presence of the migrating tumor cell (actin; Figure 1E). Metastatic melanoma (WM239) and breast cancer cells (MDA-MB231) transmigrated with similar kinetics such that cells were actively extravasating at 3-5 hours (Figures 1J and 1K).

MMPs, as well as serine proteases have been implicated in the migration and invasion of cancer cells. To investigate the involvement of selected classes of proteases during the transendothelial migration of both MDA-MB231 and WM239 cells, we first measured the degree of transendothelial migration of the cell lines in the presence of the commonly used broad-spectrum protease inhibitors ilomastat (MPi) and aprotinin (Ap). Both metalloproteinase and serine proteinase inhibitors significantly reduced the number of spreading cells at five hours by at least 35%, suggesting that both metalloproteinases and serine proteinases are involved in transendothelial migration (Fig. 1L). A dose dependent inhibitory effect on transendothelial migration was attained using 5, 10 and 20 μM MPi (not shown). No detrimental effects were detected on tumor or endothelial cells treated with 20 μM MPi using toxicity assays (MTT cell proliferation assay; not shown). 

To more specifically test the importance of MMP function we used recombinant tissue inhibitors of metalloproteinases (TIMPs; Figure 1M). Both TIMP-1 and -2 significantly blocked the transendothelial migration of both lines by approximately 35%. We again demonstrated that various concentrations of the reagents were not toxic before proceeding (not shown). 

**Figure 2:**
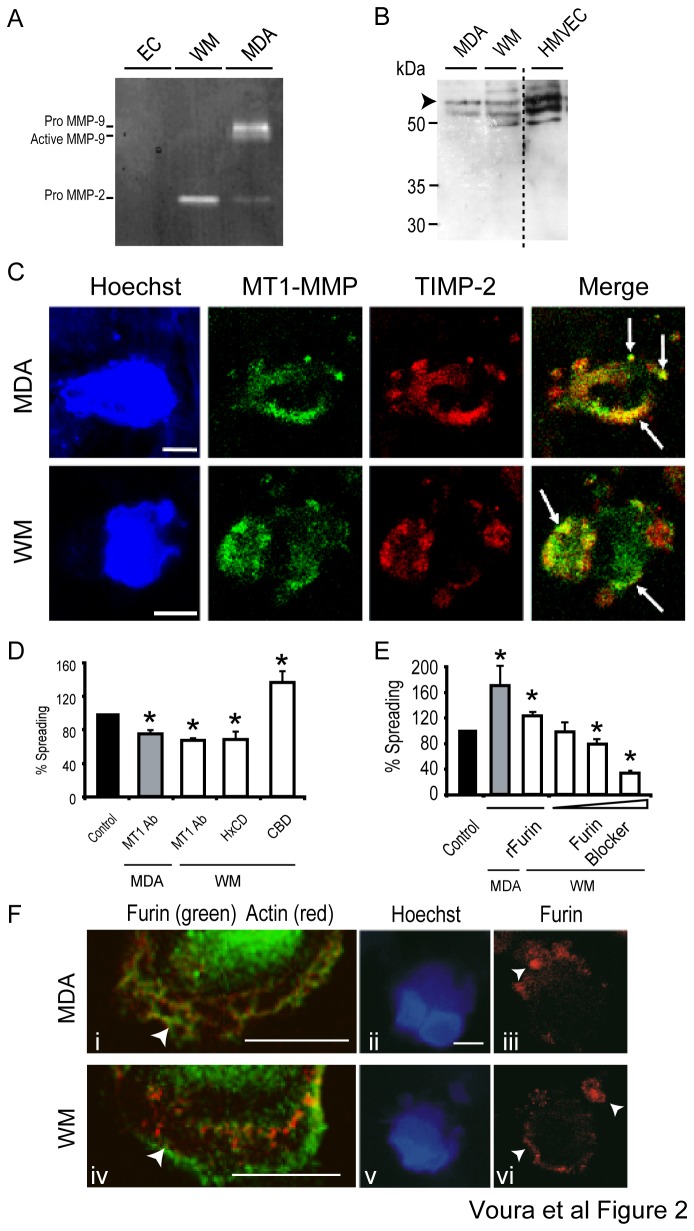
The involvement of trimolecular complex components in the process of tumor cell transendothelial migration. (A) Zymogram of serum-free, 24 hour conditioned media samples from EC, WM239 (WM) and MDA-MB231 (MDA). (B) Western blot of total cell lysates from MDA-MB231 (MDA), WM239 (WM) and EC (HMVEC) cultured in EC media stimulated with TNFα (10 ng/mL) show the expression of MT1-MMP. The arrowhead indicates the location of full length MT1-MMP. The dotted line represents an excised lane. (C) Tumor cells, with blue cell tracker (lighting up the entire cell cytoplasm and contours) and Hoechst (lighting up the nuclei) were incubated on EC (no blue cell tracker, but blue nuclei, which are not seen in this image) for 3 hours. Both tumor and EC cells were stained for actin (blue in both endothelial and tumor cells). Note that there are three ‘blue’ signals – the nuclei and actin of both tumor and endothelial cells, as well as the tumor cell cytoplasm. The upper panel shows 1 μm sections of a non-permeabilized seeking MDA-MB231 cell, the lower panel shows a non-permeabilized migrating WM239 cell labeled with MT1-MMP (green) and TIMP-2 (red). Arrows in the merged image indicate co-localization (yellow). Bars=10 μm. (D) Three reagents were tested to study trimolecular complex involvement during MDA-MB231 (gray bar) and WM239 (white bars) transendothelial migration. HxCD (1.6 mM) and the MT1-MMP mAb (MT1 Ab; 15 μg/mL) inhibited a 5-hour transendothelial migration assay. CBD (12.5 μM) stimulated melanoma extravasation at 3 hours. The relative control is the black bar. (E) Recombinant furin convertase (rFurin; 71 U/mL) stimulated transendothelial migration of MDA-MB231 (gray bar) and WM239 (white bars) cells at 3 hours. Increasing amounts of a furin inhibiting peptide decreased WM239 transendothelial migration. Increasing concentrations were 50, 100 and 200 mM. Error bars=mean±S.E.M. (F) Lamellipodia from MDA-MB231 (upper panels) and WM239 (lower panels) express furin in 1 μm confocal sections when cultured alone on Matrigel. Furin (green) localized to leading lamellipodial edges (arrowhead) with actin (red) (i and iv). Seeking Hoechst and blue cell tracker labeled (blue) MDA-MB231 (ii and iii)) and WM239 (v and vi) in 1 μm confocal sections. These images, taken on non-permeabilized transendothelial migration samples, show furin (red) at the cell surface and on blebs. Arrows described in the Results. Bars=10 μm.

Zymography analysis of serum-free conditioned media from the cell lines used in the transendothelial migration assay indicated that in isolation, endothelial cells did not express detectable levels of gelatinases (MMP-2 and MMP-9), MDA-MB231 cells produced detectable levels of pro-MMP-2 and pro and active MMP-9, whereas WM239 cells only had detectable levels of pro-MMP-2 (Figure 2A). Western blotting indicated that the full-length proenzyme species of MT1-MMP was produced by endothelial cells, as well as both the MDA-MB231 and WM239 cell lines (Figure 2B).

### The involvement of trimolecular complex components in the process of tumor cell transendothelial migration

MT-MMPs are implicated in the migration and invasion of tumor cells [44]. MMP-2 is a soluble MMP, which is activated at the cell surface by a trimolecular complex that is most commonly composed of MT1-MMP, TIMP-2 and pro-MMP-2. We used immunofluorescence staining to examine the expression of such an MT1-MMP/TIMP-2 association on the surface of tumor cells during transendothelial migration (Figure 2C). Both MT1-MMP and TIMP-2 were found on tumor cell surfaces and membrane protrusions during transendothelial migration. Arrows in the merged images point to sites of co-localization between MT1-MMP and TIMP-2. Such localization was most apparent at the late seeking to early migratory stages, suggesting that the trimolecular complex may be involved in the invasive process. 

The involvement of trimolecular complex components in the process of transendothelial migration was further examined by the addition of specific bio-modulators. Using WM239 cells, the requirement for MMP-2 activity during tumor cell extravasation was evaluated using recombinant CBD (collagen binding domain protein) [7] and MMP-2 HxCD (hemopexin C domain protein) [45], which are known to exert stimulatory and inhibitory effects on pro-MMP-2 activation, respectively (Figure 2D). Recombinant HxCD reportedly prevents the binding of pro-MMP-2 to TIMP-2 and the subsequent trimolecular complex-mediated activation of pro-MMP-2. Recombinant CBD, on the other hand, stimulates pro-MMP-2 activation by displacing pro-MMP-2 from cell surface-associated collagen, thus providing an extra pool of pro-MMP-2 that can be activated by the MT1-MMP/TIMP-2 pathway involving the formation of the trimolecular complex. WM239 extravasation was inhibited by 35% with the HxCD, peptide and reciprocally, stimulated by 40% with the CBD peptide suggesting the importance of active MMP-2 during transendothelial migration. Interestingly however, neither recombinant domain affected the migration of MDA-MB231 cells suggesting that this cell line does not utilize MMP-2 for transendothelial migration (Figure 2D). 

Next, we investigated the role of MT1-MMP by adding a function blocking MT1-MMP monoclonal antibody. This antibody inhibits the MMP-2 processing ability of MT1-MMP [43]. Typical actin staining and trypan blue exclusion in the presence of this antibody suggested it was not toxic to the tumor cell lines. In the presence of anti-MT1-MMP we observed a 35% reduction in the spreading of both WM239 and MDA-MB231 cells (Figure 2D). This suggests that while the trimolecular complex (MT1-MMP and MMP-2 activity) played a role in WM239 transendothelial migration, MT1-MMP might behave as a protease in its own right during MDA-MB231 cell transmigration. 

Proprotein convertases are suggested to regulate pericellular MMP activation. MT1-MMP can be activated by furin, and the process of this activation has been shown to occur both intracellularly or at the cell surface [9,13,14]. We asked whether the addition of recombinant furin augments tumor cell transendothelial migration and whether a furin-specific peptide inhibitor [13,46] blocks extravasation. Recombinant furin significantly increased both WM239 and MDA-MB231 cell transendothelial migration (Figure 2E), and the furin inhibitor [47] produced a significant and concentration-dependent decrease in WM239 cell transmigration. The cytotoxicity of these reagents was ruled out using trypan blue exclusion. In order to determine whether furin was present at the cell membrane, we established the surface distribution of furin by immunofluorescence. Cells plated directly on Matrigel expressed endogenous furin (green) intracellularly as well as on the leading lamellipodial edge (arrows; Figure 2F, left panels). Images were also taken of non-permealized cells during the process of transendothelial migration. These tagged tumor cells expressed furin (red) on the cell surface during extravasation (arrows; Figure 2F right panels). Collectively, these data highlight furin functionality at the tumor cell surface during transendothelial migration.

### The association of cell adhesion molecules and MMPs during tumor cell transendothelial migration

**Figure 3:**
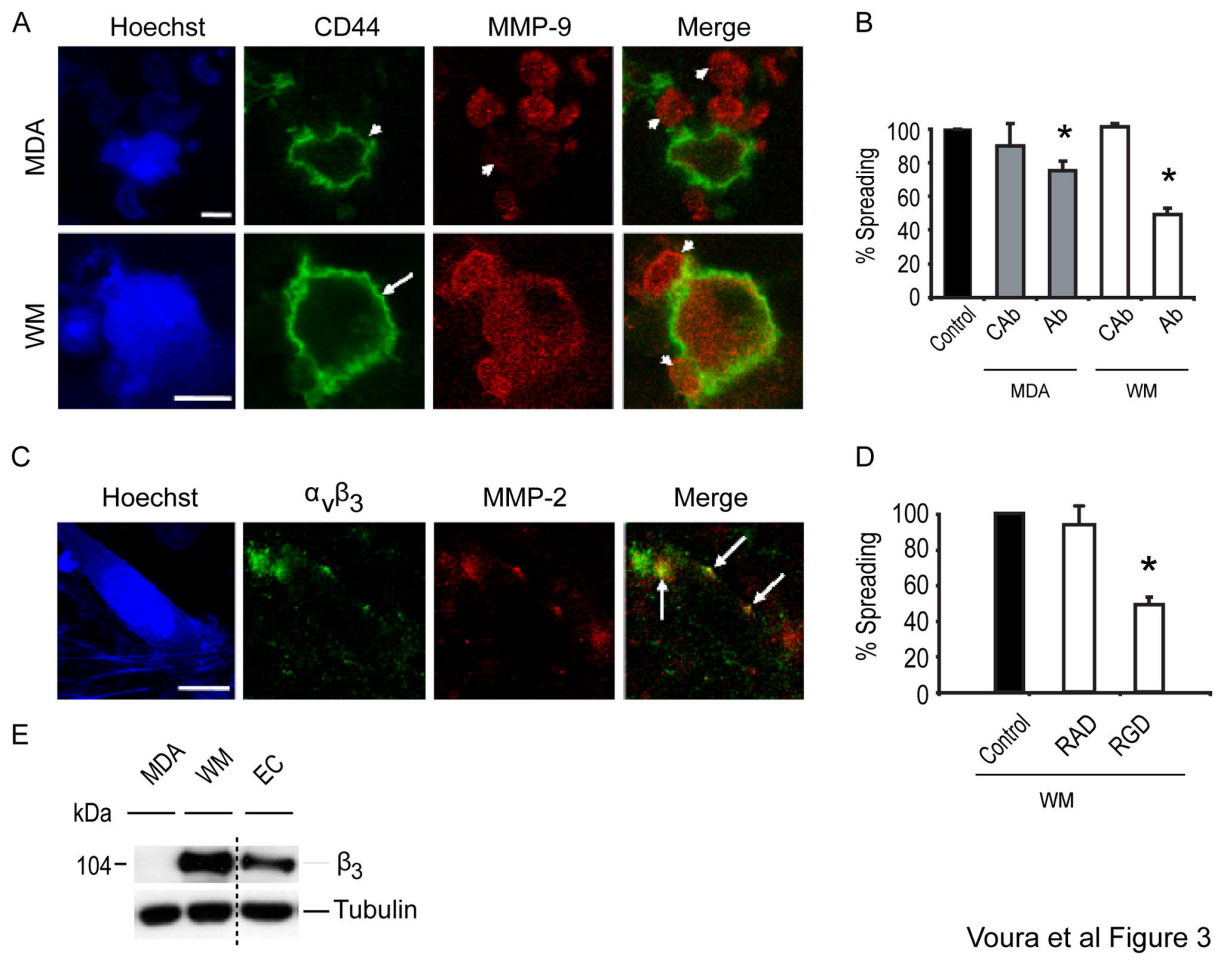
The association of cell adhesion molecules and MMPs during tumor cell transendothelial migration. (A) Blue cell tracker and Hoechst labeled tumor cells (bright blue cells and nuclei) incubated on EC for 3 hours. Both tumor and EC were stained for actin (pale blue). 1 μm sections through a seeking MDA-MB231 cell (upper panel) and a seeking WM239 cell (lower panel) labeled for CD44 (green) and MMP-9 (red) are shown. Arrowhead and arrow point to CD44 in heterotypic tumor cell-endothelial cell contacts or on the cell surface, respectively. Arrowheads also indicate little cell surface, or abundant MMP-9 staining of blebs (merged images). Note that the blebs also show circumferential actin staining in blue. Bars=10 μm. (B) Inhibitory MMP-9 mAb (Ab; 15 μg/mL) used to block MDA-MB231 (gray bars) and WM239 (white bars) extravasation. (C) 1 μm confocal sections of a blue cell tracker and Hoechst labeled WM 239 cell (bright blue cells and nuclei) incubated on EC for 3 hours and undergoing the spreading stage of transendothelial migration. Experiments were subjected to labeling of actin in both tumor and EC cells (pale blue). Labeling of α_v_β_3_ (green) and MMP-2 (red). Arrows indicate spectral co-localization (yellow) in merged image. Bars=10 μm. (D) An α_v_β_3_-specific blocking cyclic peptide (RGD; 90 μM) reduced WM239 melanoma extravasation compared with a non-blocking cyclic RAD peptide (RAD; 90 μM) as shown in the white bars. The black bar is the relative control with no peptide. (E) Western blot of total cell lysates from MDA-MB231 (MDA), WM239 (WM) and EC cultured in EC media with TNFα (10 ng/mL) show the expression of the β_3_ integrin subunit. Tubulin is a protein loading control. The dotted line represents an excised lane.

Potential mechanisms for recruiting MMPs to the cell surface involve not only membrane type metalloproteinases but also cell surface adhesion molecules. CD44 is reported to anchor and localize active MMP-9 to cellular invadipodia at the cell surface, thus promoting cell motility and invasion [27–29]. With both WM239 and MDA-MB231 cells undergoing transendothelial migration, MMP-9 was expressed at cell surface, but was also expressed in tumor cell ‘blebs’ (arrowheads; Figure 3A). CD44 and MMP-9, however, did not co-localize on these tumor bleb structures. The activity of MMP-9 in tumor cell extravasation was investigated using a function-blocking antibody [48]. Addition of this antibody led to significant reductions in spreading cells (30-50%) for both MDA-MB231 and WM239 cells when compared to inhibition by a previously identified non-blocking control antibody [40] (Figure 3B). Together, these results suggest that CD44 is not involved in the effects of MMP-9 on transendothelial migration. Interestingly, in light of the zymogram results showing no MMP-9 by either the EC or the WM239 cell line alone (Figure 2A), the confocal data suggest that MMP-9 is perhaps induced when these cells are cultured together. 

Since we did observe CD44 on the surface of the tumor cells during transendothelial migration we asked whether CD44 complexes with MT1-MMP on the leading edge of extravasating cells [30,31]. We first examined the localization of MT1-MMP and CD44 on the surface of WM239 tumor cells plated directly on Matrigel with no endothelial cell layer, and found that these molecules did co-localize (not shown). However, MT1-MMP and CD44 failed to co-localize at the cell surface of tumor cells undergoing transendothelial migration (not shown). 

We next examined whether α_v_β_3_ contributes to pericellular proteolysis during transendothelial migration. α_v_β_3_ has been shown to localize MMP-2 to the surface of invasive cells [16]. Western blots for the β_3_ subunit indicated that the WM239 cells, and not the MDA-MB231 cell line, expressed the integrin subunit suggesting that only the WM239 cells possess α_v_β_3_ (Figure 3E). The localization of α_v_β_3_ (green) and MMP-2 (red) were examined during the transendothelial migration of WM239 cells (Figure 3C). As shown previously, α_v_β_3_ was found on the surface of migrating WM239 cells including cell surface blebs and in tumor cell-endothelial cell junctions [42]. Here we observed that MMP-2 localized at the leading edges and also at discrete areas of the cell surface, and that MMP-2 and α_v_β_3_ co-localized during transendothelial migration (arrows; Figure 3C). The cyclic RGD peptide is known to functionally block α_v_β_3_-mediated interactions with vitronectin and MMP-2 [16,49]. In the presence of this blocking peptide we observed a reduced level of transendothelial migration compared to that observed in the presence of the cyclic RAD control peptide or with no peptide at all (Figure 3D). 

### Matrix digestion occurs during transendothelial migration

**Figure 4:**
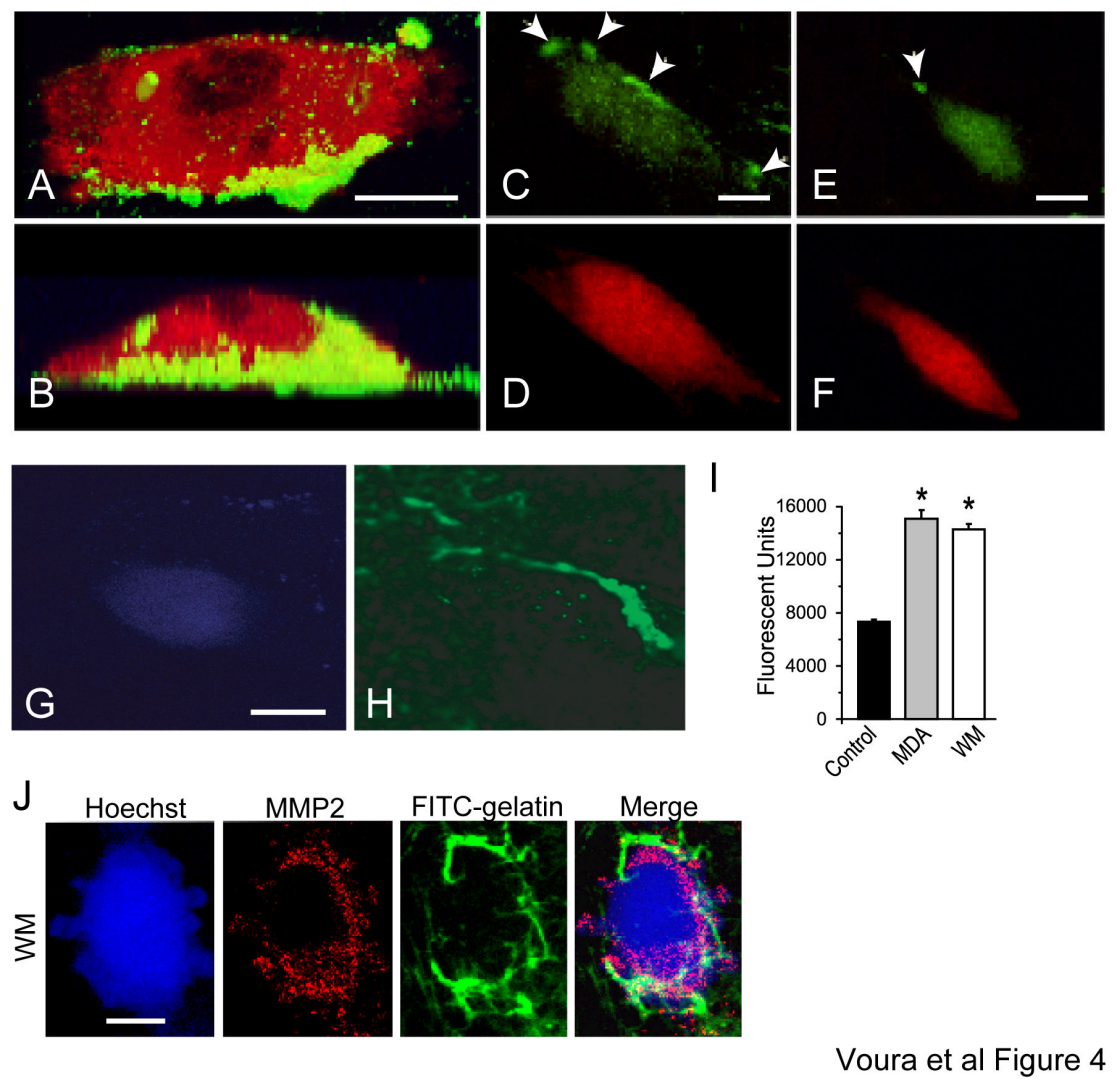
Matrix digestion occurs during transendothelial migration. A three-dimensional optical rotation of a DiI labeled MDA-MB231 cell (red) from the top (*A*) and side (*B*) as it digested and internalized extracellular matrix (green). Substrate digestion observed during extravasation (*C*-*F*). An orange cell tracker labeled WM239 (red; *D* and *F*) imaged by epifluorescence. Digestion of fluorogenic substrate is seen around cell contours of migrating and spreading cells (green; *C* and *E*). (G) A blue cell tracker and Hoechst labeled WM 239 cell (bright blue cell and nucleus) incubated on EC for 3 hours and undergoing the spreading stage of transendothelial migration. (H) Digestion of FITC-labeled fluorogenic substrate is seen around cell contours (green). Bar=10 μm (I) MDA-MB231 breast cancer cells (gray bar; MDA) and WM 239 melanoma cells (white bar; WM) digest FITC gelatin as measured by a fluorescence plate reader in serum-free conditions relative to a media only control (black bar). Error bars=mean±S.D. (J) 1μm confocal section of a Hoechst and blue cell tracker (blue) tagged WM239 cell with surface MMP-2 (red) and corresponding matrix digestion as revealed by the fluorescence signal given off from the fluorogenic substrate incorporated in the Matrigel (green). Native substrate prior to enzymatic digestion, as well as without cells provides no detectable confocal fluorescence signature due to quenching of the FITC label on the substrate. Bar=10 μm.

The observation that both serine and metalloproteinase inhibitors reduced transendothelial migration indicated that proteolysis is involved in the process. To obtain further evidence of tumor cell-mediated extracellular matrix digestion, cells were first seeded on Matrigel mixed with a fluorogenic gelatin substrate. Notably, this fluorogenic substrate, or FITC-conjugated gelatin, produces no fluorescence signal unless enzymatically cleaved. The substrate is manufactured such that the density of the FITC molecules conjugated to the substrate causes the FITC signal to be quenched unless the gelatin is digested. Confocal analysis of tumor cells plated directly on this matrix revealed zones of pericellular digestion (Figure 4A and 4B). The majority of matrix digestion occurred at the cell surface of both WM239 and MDA-MB-231 cells (not shown). Some of the cleaved gelatin was internalized by the cells and could be seen localized to the endosomes (not shown). 

Next, we visualized matrix digestion during the process of transendothelial migration. Focused proteolysis was observable by epifluorescence (arrowheads; Figures 4C-4F) and confocal (Figures 4G and 4H) microscopy. Furthermore, the surface expression of MMP-2 also closely corresponded to the pattern of ECM digestion (Figure 4J). These data provided evidence that matrix digestion by the tumor cells is largely concentrated to the cell periphery and is associated with the cell surface localization of an MMP. To have an idea of how well the tumor cells digest the FITC-conjugated gelatin, we plated cells directly on the matrix and compared the resulting fluorescence intensity over background – or the matrix without cells (Figure 4I).

### The migratory stage is most dependent on protease activity

**Figure 5:**
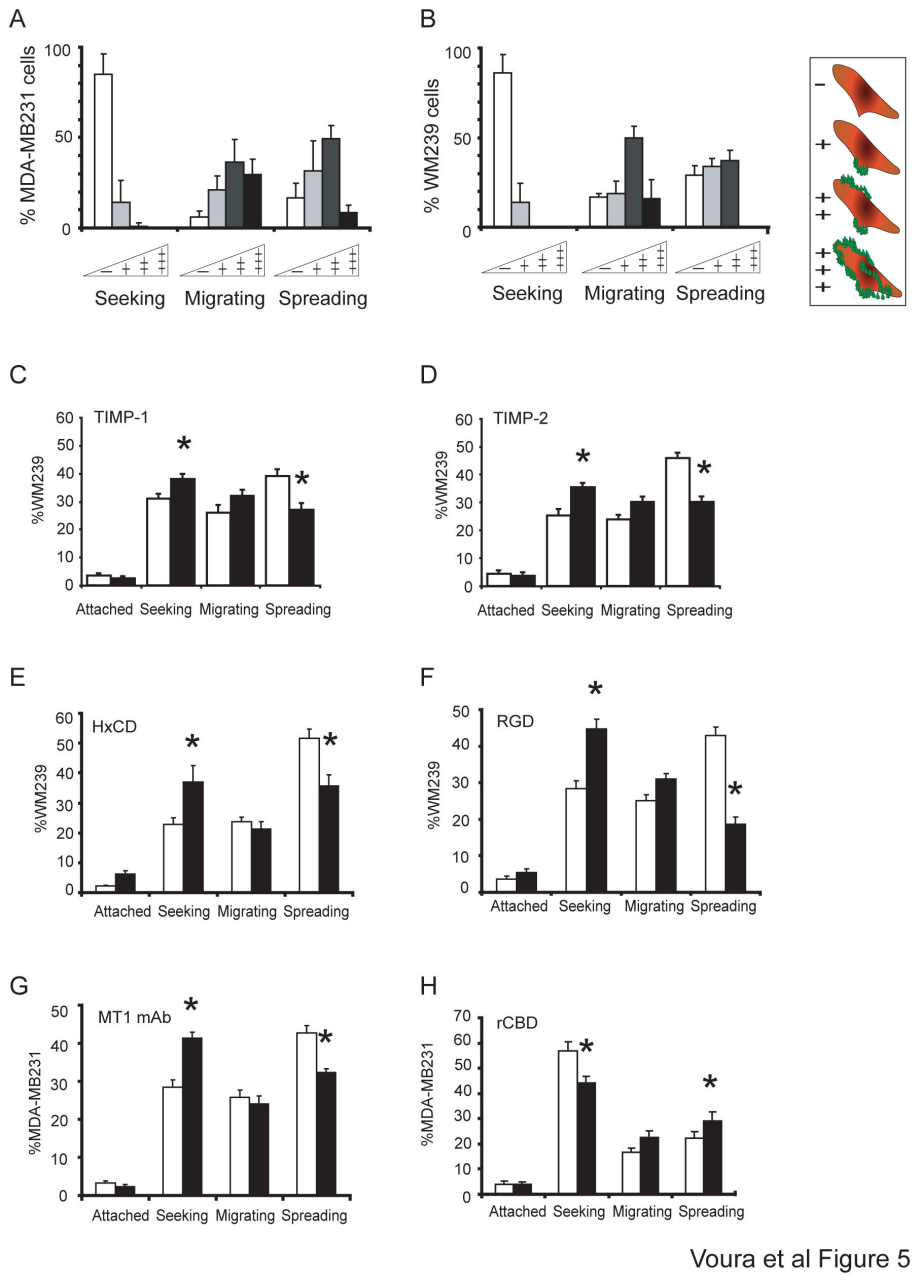
The migratory stage is most dependent on protease activity. Patches of FITC-matrix digestion were scored for both MDA-MB231 (A) and WM239 (B). “-“ = no digestion around cell contours, “+” = single patch of digestion, “++” = patchy digestion and “+++” = digestion around the entire cell contour. The cartoon panel on the right represents the scoring scheme. Error bars=mean±S.E.M.. Percent WM239 cells at each stage of extravasation upon treatment with (C) TIMP-1 and (D) TIMP-2. Percent WM239 cells at each stage of extravasation upon treatment with (E) HxCD or (F) RDG peptide. Percent MDA-MB231 cells at each stage of extravasation upon treatment with the (G) MT1-MMP blocking antibody and (H) CBD. In graphs C-H, treated cells (black bars) are compared to untreated controls (white bars). Error bars=mean±S.E.M..

To determine the stage of transendothelial migration most dependent on substrate proteolysis, we quantified fluorogenic matrix digestion using epifluorescence microscopy with cells in each stage of the process. The degree of digestion around the tumor cell contours was scored from – to +++ as indicated in Figure 5. Endothelial cell monolayers cultured without tumor cells on the matrix showed only limited digestion of the substrate (not shown). The most robust matrix digestion was first evident around the tumor cells at the migrating stage for both cell types during transendothelial migration (Figures 5A and 5B). To further assess the role of metalloproteinases by stage of extravasation, we added the biomodulators to the system and carefully examined the number of cells in each step. When TIMP-1 or TIMP-2 were added to the assay, both significantly increased the number of cells held in the seeking phase and reduced the number of spreading cells (Figure 5C and 5D). The HxCD (Figure 5E) and RGD peptide (Figure 5F) reduced the number of spread WM239 cells. The MT1-MMP blocking antibody (Figure 5G), similarly affected these stages in MDA-MB231 cells. In contrast, the recombinant CBD peptide that increased the ability of the tumor cells to undergo transendothelial migration, reduced the number of seeking MDA-MB231 cells while increasing those of spreading cells (Figure 5H). These results indicate that the stage of tumor cell extravasation most dependent on the activity of MMPs and their associated cell surface adhesion molecules is the transition from the seeking to the migrating phases. Thus, the onset of migration was most impeded by the failure to cleave matrix and tumor cells remained trapped above the endothelium.

## Discussion

**Figure 6:**
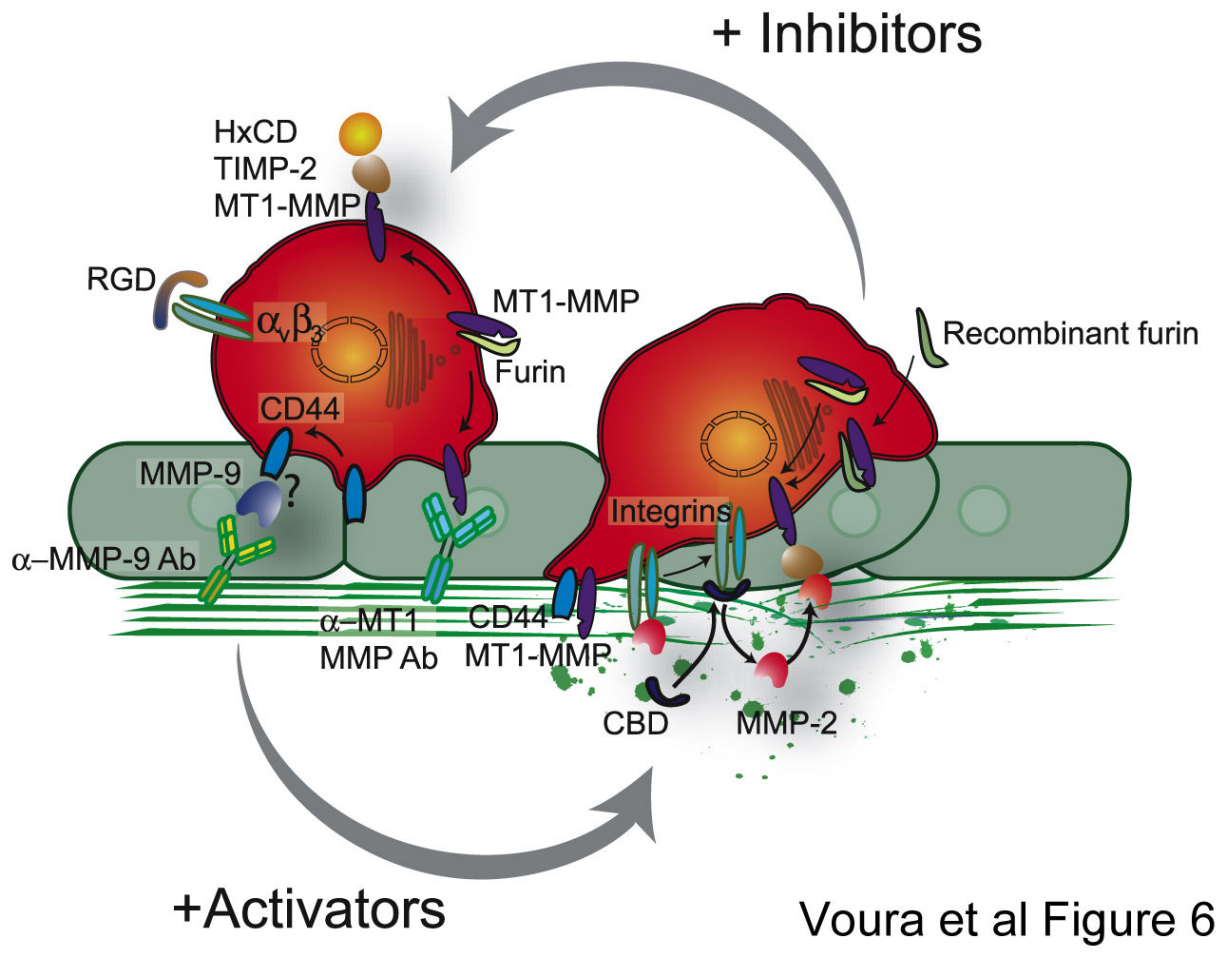
Model depicting the redistribution, complex formation and activation of MMPs during tumor cell transendothelial migration. This model indicates the sites of action of inhibitors, cell surface anchoring proteins and activators during the process of transendothelial migration in this study.

Using an *in vitro* model, this study provides biochemical and microscopic evidence for some key components of the proteolytic interface that operates at the tumor cell surface during transendothelial migration (Figure 6). Distinct classes of molecules cooperate to generate a unified mechanism to bring about precisely regulated and limited pericellular proteolysis. Especially important is the requirement of proteolysis at the migrating stage when cells actively cross the endothelial barrier. Although the literature supports a role for proteinases in augmenting, and protease inhibitors in suppressing, metastasis [50–53], the requirement of proteolysis during extravasation has been questioned [36,37]. In general, experimental systems that have addressed extravasation have been unable to map the complex molecular interplay of the proteinases, their inhibitors and cell adhesion molecules involved in the process. Our study begins to address these issues and provides a framework for functional proteolysis at the cell-matrix interface during cancer cell transendothelial migration.

 The transendothelial migration assay is a model of tumor cell extravasation, which enables us to question the involvement and interaction of proteases and cell adhesion molecules during the process of metastasis. Tumor cells plated above an endothelial monolayer are scored according to the stage of passage through this endothelium (Figure 1). Transmigration is active approximately five hours after cell attachment. Synthetic inhibitors of serine and metalloproteinases, as well as recombinant TIMPs, exerted a significant reduction in the ability of cells to transmigrate. The involvement of MMPs and cell adhesion molecules was explored through the use of confocal microscopic visualization of molecules and proteolysis, and functionally by the use of specific biomodulators (Figures 2-5). 

Co-localization of MT1-MMP and TIMP-2 was suggestive of the formation of the trimolecular complex with MMP-2 (Figure 2C). Inhibition by the MT1-MMP mAb revealed the importance of MT1-MMP for the extravasation of both cell lines. Intriguingly, recombinant HxCD and CBD peptides only affected WM239 melanoma cell transendothelial migration (Figure 2D). While, zymogram analysis gave evidence that only the WM239 cell line secreted MMP-2 (Figure 2A). Hence, MT1-MMP activity was important for transendothelial migration, but active MMP-2 bound to MT1-MMP, as part of the trimolecular complex, was not necessary for both cell lines. Inhibition of the formation of active MT1-MMP was also investigated by altering the activity of furin, a proprotein convertase capable of processing MT1-MMP, an event shown to be integral to overall tumorigenesis [11]. This demonstrated the importance of active MT1-MMP in our system since the transendothelial migration of WM239 cells was reduced in the presence of a furin inhibitor (Figure 2E). This inhibitor, however, did not affect migration of MDA-MB231 cells, which raises the possibility of furin-independent MT1-MMP activation in this cell line [9,13,14,54]. Notably, the addition of recombinant furin augmented the transendothelial migration of both cell lines indicating the importance of MT1-MMP to enhance the extravasation of both WM239 and MDA-MB231 cells, albeit in a somewhat different manner in the two lines. These results are supported by the findings of Sabeh et al. [55], who presented data documenting the importance of MT1-MMP for tumor cell migration through the ECM.

 Most extracellular matrix degrading enzymes are not membrane-anchored molecules and depend on their association with cell surface protein complexes for surface localization. We used confocal microscopy to visualize enzymes and linking molecules, and modulated transendothelial migration with an array of modifying reagents (Figure 5). MMP-2 was present at the cell periphery and on lamellipodia, co-localized with α_v_β_3_, suggesting the integrin is involved, directly or indirectly, in linking MMP-2 to the cell surface (Figure 3C). It was shown previously that α_v_β_3_ is located on melanoma cells and in tumor cell-endothelial cell contacts [42]. Furthermore, a α_v_β_3_-blocking RGD peptide has been reported to reduce the percentage of spreading WM239 cells [49] and this peptide abrogated both α_v_β_3_-mediated interactions with ECM and α_v_β_3_ ligation to MMP-2 [16]. MMP-2 and α_v_ integrins were linked to ovarian carcinoma cell invasion of endothelial cells *in vitro* [20]. We suggest, therefore, that RGD the peptide likely disrupts the α_v_β_3_/MMP-2 complex and inhibits matrix digestion at the migrating stage of extravasation (Figure 3D). Confirming these results, we detected the expression of the β_33_ integrin subunit by the WM239 cell line (Figure 3E).

In contrast, the co-localization of CD44 with MMP-9 was not detected (Figure 3A). Unlike reports by Yu et al. [28,29], Bourguignon et al. [27] and Legrand et al. [56] we, like Dolo et al. [57], found MMP-9 expression predominantly on ‘bleb’ structures (Figure 3A). We demonstrated that an MMP-9 mAb inhibited the transendothelial migration of both tumor lines (Figure 3B) and propose that MMP-containing blebs from tumor cells augment local MMP levels. Poste and Nicolson [58] showed that bleb components from metastatic cells confer aggressive qualities upon poorly metastatic cells, while others have found that blebs contain both serine proteinases and gelatinases [59–62]. Further, it is possible that MMP-9 activity is activated by the collagen-binding domain (CBD) of MMP-2, or via MMP-9 dimerization through the hemopexin domain in our system [63,64]. However, the function of the observed bleb structures and exactly how MMP-9 contributes to, and is activated and expressed during, tumor cell transendothelial migration remains unresolved and will form a subject for further investigation. 

Transendothelial migration is a dynamic process, with one stage leading to the next. We observe that, the presence of proteinase inhibitors and biomodulators led to an altered stage distribution: a decrease in late stage spreading cells, and an increase in early-stage seeking cells, with migrating cell numbers remaining unchanged (Figure 5C-5H). Co-localization of transmembrane molecules with extracellular proteinases, suggesting complex formation at the cell surface, was most apparent during migration. Furthermore, most robust digestion was visualized at the migrating stage (Figure 5A and 5B). Collectively, this evidence indicates that the transition between the seeking and migrating stages is particularly reliant upon proteolysis. The tumor cells remain trapped above the endothelium when proteolytic digestion of the underlying ECM is reduced. These findings correlated to the results obtained by Leroy-Dudal et al. using ovarian carcinoma cells and human endothelial cells in culture [20]. A slower entry into, or exit from the migrating stage, gives rise to altered tumor cell numbers at the seeking and spread stages. It is important to note here, that the detectable proteolytic digestion using our assay was, therefore, localized to and active on the ECM (Figure 4). We did not find evidence of the generalized digestion, or weakening, of the endothelial monolayer either in the presence of the protease inhibitors, or in regions removed from the tumor cells [38]. Indeed, the purpose of the actin staining in each experiment was to ensure that the endothelial monolayer was intact and that connections between the endothelial cells were maintained. The endothelial cells were also plated at a high density to avoid ‘holes’ that can result due to contact inhibition. However, since inhibitors of both serine and metalloproteinases significantly inhibited extravasation (by 35-40%) (Figure 1L), but did not totally abrogate it, suggests additional processes are at work. Some of these possibilities include; other matrix-digesting enzymes or complexes are involved, intimate signaling between the tumor and endothelial cells is required, or that a proteinase-independent mechanism of tumor cell migration may also operate [35,65–68]. 

In summary, we highlight the importance of MT1-MMP and MMP-9 activities for both cell lines tested (WM239 and MDA-MB231) in this study, while stressing that MMP-2 played an additional role with one (WM239). The commonality between both lines, however, was the stage of extravasation that was particularly reliant upon proteolysis – the interaction of the tumor cells with the basement membrane underlying the endothelium. From our results, therefore, we envision a dynamic process where cell adhesion molecules and MT-MMPs redistribute to the invading edge of tumor cells during transendothelial migration, providing a means to both anchor and activate MMPs, and by this means localize ECM cleavage. Tumor cells probe the endothelial junctions and then use proteolysis to complete the penetration of the basement membrane underlying the microvasculature. We suggest that in the absence of proteolysis, probing/seeking behavior is retained, but is non-productive, in that invadopodia can form but cell extravasation is reduced (Figure 6). An orchestration of the physical presence, subcellular location, and activity of multiple protein classes brings about extravasation, a complex biological function essential for metastasis. A greater understanding of this process is critical as the field sorts out the various biological roles of metalloproteinases in an effort to strike a balance between general patient health and anti-cancer therapy of this diverse family of enzymes [1,2].
